# Oral glucose tolerance test and continuous glucose monitoring for gestational diabetes diagnosis: a survey study of women and health care professionals

**DOI:** 10.1007/s00404-023-06949-2

**Published:** 2023-02-05

**Authors:** Daria Di Filippo, Justine Darling, Melissa Han Yiin Chang, Amanda Henry, Alec Welsh

**Affiliations:** 1 School of Women’s and Children’s Health, University of New South Wales, Sydney, NSW Australia; 2 Diabetes Clinic, Royal Hospital for Women, Sydney, NSW Australia; 3 Department of Women’s and Children’s Health, St George Hospital, Sydney, NSW Australia; 4 Department of Maternal-Fetal Medicine, Royal Hospital for Women, Locked Bag 2000, Barker Street, SydneyRandwick, NSW 2031 Australia

**Keywords:** Oral glucose tolerance test, Gestational diabetes mellitus, Diagnosis, Acceptability, Perspectives

## Abstract

**Aims:**

The oral glucose tolerance test (OGTT), used for gestational diabetes mellitus (GDM) diagnosis for over 65 years, has poor acceptability and tolerability. Continuous glucose monitoring is being considered as potential alternative. The aim of our study was to formally assess women’s and health care professionals’ perception of both tests as diagnostic tools for GDM.

**Methods:**

Participants in a pilot study on continuous glucose monitoring for GDM diagnosis were invited to fill two questionnaires, each of 6 Likert-scale and one optional open-ended question. A range of healthcare practitioners were also invited to fill a questionnaire of 13 Likert-scale and 7 optional open-ended questions.

**Results:**

Sixty women completed the OGTT and 70 the continuous glucose monitoring questionnaire. OGTT was reported as poorly acceptable. Continuous glucose monitoring was described as significantly more tolerable (81% vs 27% 5/5 general acceptability rate, *p* < 0.001); ninety-three percent of the participants would recommend it for GDM diagnosis. Thirty health care professionals completed the survey. Most of them (73%) had confidence in OGTT as a diagnostic test for GDM with 66% raising some concerns. Doubts on continuous glucose monitoring were raised in terms of costs, accessibility and accuracy for GDM diagnosis due to “lack of evidence”.

**Conclusions:**

Continuous glucose monitoring was substantially better tolerated for women than OGTT. Current lack of evidence for diagnostic accuracy for GDM underlines the need for studies on correlation between continuous glucose monitoring parameters and pregnancy outcomes to strengthen evidence for its use as diagnostic test for GDM.

**Supplementary Information:**

The online version contains supplementary material available at 10.1007/s00404-023-06949-2.

## What does this study add to the clinical work

**Table:** 

CGM is significantly more acceptable for women than OGTT for GDM diagnosis. HCP professionals consider CGM significantly less acceptable than women and ask for further evidence of the correlation with perinatal outcomes.	

## Introduction

The oral glucose tolerance test (OGTT) was first introduced for gestational diabetes (GDM) diagnosis in 1957 [1]. Its pre-analytical, analytical and post-analytical limits have been widely described in the literature [2]. Low acceptability due to the OGTT logistics as well as side effects of the glucose beverage have also been reported [3].

OGTT thresholds have been changed several times over the last decades: the latest change was brought by the International Association on Diabetes and Pregnancy Study Groups (IADPSG) in response to the Hyperglycemia and Adverse Pregnancy Outcome (HAPO) study [2]. As a result, the number of women diagnosed with GDM has increased dramatically although without significant improvements in maternal or fetal/neonatal outcomes [4–6]. In twin pregnancies, this could be due to higher OGTT thresholds related to negative outcomes than those recommended by IADPSG [7]. GDM incidence is also increasing due to advancing maternal age, increased use of assisted reproductive techniques and rates of obesity [8, 9].

Some GDM risk factors, such as obesity and family history of GDM/Type 2 diabetes mellitus (T2DM) are also recognized as long term outcomes of GDM for both mothers and newborns. This demonstrates not only an intergenerational influence but also an expanding cascade effect [10, 11]. As obesity and T2DM are climbing the list of the most common causes of death worldwide, being able to correctly identify, prevent and manage GDM, becomes an ideal strategy to improve the health of current and future generations [12, 13].

A wide range of biomarkers have been explored as potential alternative to the OGTT, although none has been demonstrated to be sufficiently accurate for GDM diagnosis [14]. The main limit to research is the lack of a gold standard to rely on, apart from the OGTT with its above-mentioned limits. Therefore, any investigation aiming to prove superiority of diagnosis against the current OGTT has only the OGTT as the benchmark.

Continuous glucose monitoring, (CGM) consisting of a subcutaneous sensor which automatically tracks blood glucose levels, represents a promising alternative to OGTT. To date it has been mainly used in pregnancy for women with Type 1 diabetes mellitus (T1DM), and more recently for GDM management [15, 16]. However, there is minimal literature addressing the role of CGM as a screening test for GDM [8, 17, 18]. To our knowledge, no formal assessment of OGTT acceptability in comparison to CGM acceptability for either women or healthcare professionals (HCP) has been reported so far.

Purpose of this study was to formally assess OGTT and CGM perception of women who have undergone both tests as part of a pilot study on the use of CGM for GDM diagnosis. A secondary aim was to understand the perspective of health care professionals involved in pregnancy care regarding the use of CGM for GDM diagnosis, in terms of perceived acceptability (for patients and for them) and of reliability.

## Methods

### Participants, setting and recruitment

Women were requested to fill the questionnaires on OGTT and CGM as part of a previous pilot study evaluating the use of the Freestyle Libre Pro (Abbott, Australia) for GDM diagnosis, based in two metropolitan hospitals in Sydney. The participants (*n* = 106) were administered the surveys, as Google questionnaires, from April 2021 to April 2022, (Supplementary material 1) on the day of their OGTT, which was also the last day of a 7 day CGM monitoring period. Questionnaires were linked to a website (www.cgm4gdm.net), where women could also find more information about GDM, CGM and the study.

All the endocrinologists, midwives, obstetric consultants/trainees and diabetes educators employed within the two hospitals where the pilot study took place were requested, via email from their respective managers, to fill an online Redcap survey between the end of April to the end of June 2022. Written informed consent was obtained for all patient participants at recruitment, and was implicit for HCP participants upon reading the email, consenting to participate and filling the questionnaire (Supplementary material 2). Questionnaire results were automatically stored online in password protected accounts through Google (RTM) and Redcap (RTM). Data analysis spreadsheet were generated from the software and data integrity was verified before coding and anonymizing the questionnaires of the patients.

### Study design and approval

A semi-quantitative survey approach including multiple choice questions, open-ended/free-text questions and Likert scale questions was undertaken to explore perceived OGTT and CGM acceptability for women and health care professionals. This study was performed in accordance with the Declaration of Helsinki and was approved by South Eastern Sydney Local Health District HREC (2019/ETH04910).

### Data analysis

Women’s demographics were analyzed extensively as part of the pilot study. HCP demographics and work experience information were reviewed and summarized. Likert answers were analyzed quantitatively, and categorical variables expressed as percentages of different scores were compared between groups (women’s perception of OGTT vs CGM, and women’s vs HCP’s perception of OGTT and CGM) using *χ*^2^ test using SPSS. Values of *p* < 0.05 were considered significant. Responses to open-ended questions were grouped in themes and summarized [19].

## Results

### Women’s perception of OGTT and CGM

Sixty women completed the questionnaire on OGTT and seventy the questionnaire on CGM. The mean age of participants was 32 years, with gravidity ranging from 1 to 6 and parity from 1 to 3. Twenty-five of the 70 participants had at least one risk factor for GDM (age > 40 years, BMI > 25, high-risk ethnicity, polycystic ovarian syndrome, family history of diabetes mellitus) [20]. Table 1 summarizes women’s responses regarding the OGTT. A majority of participants (80%) found the test acceptable rating it 3–5/5. However, 20% found the test generally unacceptable (1–2/5). Having to fast for the test was rated 3–5/5 by 80% of the women. Only 10% of the participants rated the glucose beverage 5/5, and more than half (53%) found it unacceptable (1–2/5). Blood collection was described as acceptable (3–5/5) by 84% of the women. More than half of the participants (56%) found the OGTT timeframe, including the 2 h waiting period, unacceptable (1–2/5) and only 12% rated it 5/5. In terms of likelihood of recommending the OGTT to other women, only 12% of participants gave a 5/5 response and 47% of them a 1–2/5 response.

**Table 1:** OGTT acceptability for pregnant women

OGTT aspects investigated	RATING/5 *n* (%) (total *n* = 60)				
	1	2	3	4	5
General acceptability	3 (5%)	9 (15%)	16 (27%)	16 (27%)	16 (27%)
Fasting for OGTT	2 (3%)	10 (17%)	14 (23%)	18 (30%)	16 (27%)
Glucose beverage	11 (18%)	21 (35%)	9 (15%)	13 (22%)	6 (10%)
Blood collection	2 (3%)	8 (13%)	12 (20%)	13 (22%)	25 (42%)
OGTT timeframe	8 (13%)	26 (43%)	11 (18%)	8 (13%)	7 (12%)
Likelihood of recommending it to other women	15 (25%)	13 (22%)	17 (28%)	8 (13%)	7 (12%)

*OGTT* Oral glucose tolerance test

Table 2 reports women’s feedback on CGM. In terms of general acceptability, no participant found CGM unacceptable (i.e., no scores of 1–2/5) and 81% (57/70) of the women gave a 5/5 score. This is a significantly higher score than for the OGTT for which only 16/60 participants gave a 5/5 score (*p* < 0.001). For CGM insertion, wearing and removal there were no score 1 or 2/5 reported: respectively with 83% 5/5 for insertion, 81% 5/5 for wearing and 73% 5/5 for removal. When asked if they would have recommended CGM as a diagnostic test for GDM, 93% of the women responded very much likely (4–5/5), 7% quite likely (2–3/5), with no 1/5 responses. The percentage of women who would recommend the CGM for GDM diagnosis with a 5/5 score was significantly higher than for OGTT (7/60–11.7% vs 56/70–80%, *p* < 0.001).

**Table 2:** CGM acceptability questionnaire for pregnant women

CGM aspects investigated	RATING/5 *n* (%) (total *n* = 70)				
	1	2	3	4	5
General acceptability	0 (0%)	0 (0%)	2 (3%)	11 (16%)	57 (81%)
(comparison with OGTT (*n* = 60))	3 (5%)	9 (15%)	16 (27%)	16 (27%)	16 (27%)
Insertion	0 (0%)	0 (0%)	2 (3%)	10 (14%)	58 (83%)
Wearing	0 (0%)	0 (0%)	4 (6%)	9 (13%)	57 (81%)
Removal	0 (0%)	0 (0%)	6 (9%)	13 (19%)	51 (73%)
Likelihood of recommending it to other women	0 (0%)	1 (1%)	4 (6%)	9 (13%)	56 (80%)
(comparison with OGTT (n = 60))	15 (25%)	13 (22%)	17 (28%)	8 (13%)	7 (12%)

*CGM* Continuous glucose monitoring

The comments recorded by women in the free-text section of the OGTT and CGM questionnaires are reported in Supplementary material 3. Most comments on OGTT (8) were regarding the side effects of the glucose beverage which was difficult to consume within five minutes, and caused nausea, light-headedness, and general malaise. Six participants highlighted the need for an alternative test and five the challenges represented by the OGTT, which was reported to be a big inconvenience. Four women reported concerns regarding the glucose load and the sitting period of two hours encompassed by the OGTT being not representative of their lifestyle.

Regarding CGM, women mostly commented on the minimal impact on daily life (15 comments). Ten women reported diet/training tracking as being poorly acceptable, limited by a defective app and influencing their diet representing a risk of bias for the study. In terms of technical issues, two women reported minimal bleeding at site of insertion, two described the risk of the sensor getting stuck in clothes/furniture when walking, two women reported itchiness and two described the removal uncomfortable. Six women underlined their willingness to recommend this test over OGTT for GDM diagnosis. Among the free comments, two women recommended to shorten the CGM period to 3 days and two women proposed the option of allowing women to self-remove the sensor at home and sending it to the treating team.

### HCP’s perception of OGTT and CGM

Thirty HCP filled the questionnaire, including midwives (10), obstetric specialists (7), diabetic educators (6), endocrinologists (4) and obstetric trainees (3). Most (60%) were 41–60 years old, had more than 15 years of experience (50%) and had been reviewing more than 50 women diagnosed with GDM per year (66%). All considered GDM as having either a crucial (69%) or a ‘somewhat important’ impact (31%) on global health. Most HCP (73%) had confidence in OGTT as a diagnostic test for GDM, although 66% of them declared that there are some or numerous issues with the current method of GDM diagnosis.

HCP evaluation of the OGTT is described in Table 3. Less than half scored general acceptability as high (46% rating 4–5/5). More than half gave a poor rating to the glucose beverage (50% rating 1–2/5; none rating 5/5). The blood collection component was rated higher (63% rating = / > 3/5). More than half rated the timeframe as poorly acceptable (53% rating 1–2/5). Reliability was given a higher rating, with 74% giving a response ≥ 3/5, although replicability was lower with 64% giving a response ≥ 3. No significant difference was found between HCP and women’s perception of OGTT acceptability.

**Table 3:** HCP perception of OGTT

OGTT aspects investigated	RATING/5 *n* (%) (total *n* = 30)					
	Missing	1	2	3	4	5
General acceptability	1 (3%)	1 (3%)	6 (20%)	8 (27%)	13 (43%)	1 (3%)
Glucose beverage	1 (3%)	3 (10%)	12 (40%)	10 (33%)	4 (13%)	0 (0%)
Blood collection	1 (3%)	0 (0%)	10 (33%)	7 (23%)	9 (30%)	3 (10%)
OGTT timeframe	1 (3%)	6 (20%)	10 (33%)	4 (13%)	8 (27%)	1 (3%)
Reliability	1 (3%)	0 (0%)	7 (23%)	8 (27%)	9 (30%)	5 (17%)
Replicability	1 (3%)	0 (0%)	10 (33%)	11 (37%)	6 (20%)	2 (7%)

*HCP* Healthcare professionals, *OGTT* Oral glucose tolerance test

Nine HCP (32%) had had previous direct experience with one or more CGMs (8 Freestyle Libre 2; 7 Medtronic iPro2; 7 other devices). When asked if they would consider the use of CGM for GDM diagnosis, a majority (54%) responded positively (23% “absolutely yes” and 31% probably yes) while only 2 participants responded negatively.

HCP evaluation of CGM is described in Table 4. In rating CGM, all the surveyed HCP evaluated the general acceptability, insertion of the device for themselves and the women as well as wearing and removing the device (again both for themselves and the women) above 1/5. Two HCP rated the general acceptability as 2/5, and 16 (53%) as 4–5/5. Rating for HCP insertion was high (87% 3–5/5;).. HCP’s perception of CGM insertion’s acceptability for women was significantly lower than that expressed by the women themselves (20% HCP vs 81% women rating it 5/5, *p* < 0.001). Wearing was considered very acceptable (4–5/5) by 36% of the HCP cohort and poorly acceptable (2/5) by two of them. Eighty-seven percent rated the removal of CGM acceptable (3–5/5), both for them and their patients. Again, HCP perceptions regarding CGM for women were significantly less positive than that of the women themselves, for both CGM wearing (3% HCP versus 81% women gave 5/5 rating, *p* < 0.001) and removal (20% versus 73% 5/5 rating, *p* < 0.001).

**Table 4:** HCP perception of CGM

CGM aspects investigated	RATING / 5 *n* (%) (total *n* = 30)					
	Missing	1	2	3	4	5
General acceptability	4 (13%)	0 (0%)	2 (7%)	8 (27%)	10 (33%)	6 (20%)
Insertion of the device						
For yourself	4 (13%)	0 (0%)	0 (0%)	16 (53%)	5 (17%)	5 (17%)
For the patients	4 (13%)	0 (0%)	1 (3%)	13 (43%)	7 (23%)	5 (17%)
Wearing	4 (13%)	0 (0%)	2 (7%)	13 (43%)	10 (33%)	1 (3%)
Removing the device						
For yourself	4 (13%)	0 (0%)	0 (0%)	13 (43%)	5 (17%)	8 (27%)
For the patients	4 (13%)	0 (0%)	0 (0%)	13 (43%)	7 (23%)	6 (20%)

*CGM* Continuous glucose monitoring, *HCP* Healthcare professionals

The free text comments of HCPs are reported in Supplementary material 4. Most related to GDM impact, which was reported as underestimated (2), having long-term consequences on health (10) as well as impact on the healthcare system and care. Fourteen HCP commented on OGTT limits in terms of: pre/analytical/post-analytical issues (2), poor reliability/reproducibility (7), and poor acceptability for women (5). Doubts on CGM for GDM diagnosis were raised by eleven HCP in terms of costs and accessibility, accuracy due to lack of evidence for effectiveness in identifying the highest risk patients and possibility of bias due to the opportunity for patient to manipulate CGM results by exercising more, eating better, during testing week to avoid GDM diagnosis.

Almost all HCP (92%) had had previous experience with complementary biomarkers for GDM such as HbA1c and fasting blood glucose. Seven of them (27%) suggested potential biomarkers for triangulation in the relevant free-text comment: early pregnancy HBA1C to exclude frank diabetes, 28^th^ weeks HbA1c/fasting blood glucose/fetal growth rate as well as maternal weight gain in third trimester > 0.5 kg per week.

## Discussion

To the best of our knowledge, this is the first study to report and compare women’s and HCP’s perception of both OGTT and CGM for GDM diagnosis. OGTT was rated as highly inconvenient and poorly accepted by women, especially in terms of timeframe and glucose beverage. Almost half of the surveyed women would be unlikely to recommend OGTT for GDM diagnosis to other pregnant women. HCP rated OGTT as overall acceptable for patients, although most of them expressed some doubts on the current diagnostic method and wished to change the current GDM diagnostic test.

CGM was significantly more acceptable from both women’s and HCP perspectives. A significant larger proportion of women in our study would recommend CGM for GDM diagnosis to other pregnant women. Given the different nature of the two tests it was not possible to compare the acceptability of specific aspects. No women nor HCP scored any of the considered CGM aspects (general acceptability, insertion, wearing and removal) as low as 1/5.

In a recent article by Kirke about OGTT in remote Australia, patient factors limiting compliance to the OGTT were reported including women not liking the test, especially if “health-conscious” and concerned of the effect of the glucose beverage on their body, but also women experiencing nausea and vomiting and having no time availability to complete the test or possibility to overcome the logistical limits (having to attend the test in a laboratory) [21]. All these perspectives were confirmed in the free-text section of our study by the participating women, who also reported concerns over the ability of the OGTT to reflect their daily glucose metabolism, as being poorly reflective of their usual activities and meals.

Among clinician factors influencing adherence to OGTT, alternative tests offered as a compromise to reluctant women and confusion among thresholds and upcoming biomarkers were reported in the article by Kirke [3]. HCP participating in our study underlined in the free comments the several pre-analytical/analytical and post-analytical issues of the OGTT as undermining its reliability for them and among their patients.

The high acceptability of CGM described by women is consistent with previous reports on its use as a management tool for GDM [22], although this is the first study to assess the acceptability for CGM as a diagnostic test for GDM, in comparison with the OGTT. CGM has hence mainly been assessed for GDM management. The positive feedback expressed by the pregnant women participating in our study demonstrate that CGM is more acceptable than the OGTT for GDM diagnosis, regardless of their risk for GDM. In the free text-section, some participants suggested a shorter CGM period of three instead of seven days. Although most studies evaluating CGM parameters consider a 7–14 day monitoring period, a shorter duration could represent a good compromise for its use as a diagnostic test still potentially giving a good insight into glucose metabolism. The reliability of a 3-days CGM period for GDM diagnosis will need to be proven in a prospective cohort study correlating CGM data with GDM outcomes. Diet and training diary were also reported as annoying by some of our participants.

We propose that women could scan the monitor on the third day and remotely share data with their treating HCPs. Those with normal BGL profile could then cease monitoring (and be considered as having no GDM), while those identified as having labile or borderline BGL control would be asked to continue wearing the device for another 4 days. In this second part of CGM wearing, women could be requested to keep track of their diet and exercise to evaluate how their BGLs respond to their lifestyle, and identify key points for their management, such as personalized change in food/exercise quality and quantity. The opportunity to add this part of assessment as a second step would still offer a wider evaluation of glucose metabolism dynamics in those women considered at risk of having/developing GDM after three days of monitoring. This would minimize the impact of CGM monitoring as a population screening test for women, reducing the overall burden but focusing on those requiring closer evaluation.

As suggested by our participants, women could then use a pre-addressed envelope to return the CGM sensors after having scanned and removed them at home, saving women’s and HCP’s/healthcare settings’ time and resources. The opportunities for remote care offered by CGM application to diabetes in pregnancy management have previously been reported [23], and would be consistent with this strategy.

Significantly lower percentages of HCP than women rated CGM insertion, wearing and removal for their patients as highly acceptable (*p* < 0.001), although practical experience in use of CGM was limited among HCPs. Interestingly, only 32% had had previous direct experience with CGMs. The disparity between patients’ and HCP’s perception of acceptability for insertion, wearing and removal for CGM may reflect the lack of direct experience of the HCPs. The reduced perception of patient acceptability for CGM as a diagnostic test for GDM could also be influenced by what was described by Miller as “therapeutic inertia” when referring to the hesitance of HCP to adopt CGM for managing diabetic patients, mostly due to the perception of data overload and need for additional education [24]. The paucity of available literature corroborates HCP’s disorientation on CGM applicability for GDM diagnosis [8, 17, 18].

Given the relative lack of data currently available, HCPs requested a cost-effectiveness analysis of CGM use for GDM diagnosis as well as a confirmation of the correlation of CGM with GDM outcomes, in order to support its future application. This is consistent with a previous review on CGM use in GDM management by Niranjala that concluded that despite the predicted increasing prevalence of GDM, future funding for CGM diagnostic protocols will depend on strong evidence of improved outcomes through its application [25]. This has been previously described in the CONCEPTT study for CGM use in T1DM monitoring during pregnancy [26]. In the CONCEPTT study, the number of pregnant women needed to treat with CGM to prevent one newborn complication was six in terms of neonatal intensive care admission and large for gestational age and eight for neonatal hypoglycaemia, contributing to a yearly cost savings of over £9 million for the adoption of CGM over SBGM, despite a much higher cost for each single CGM use compared to SBGM [26].

Our group recently published the only pilot study assessing CGM use independently from the OGTT, using the Medtronic iPro2 CGM, where we identified 11/60 FP (18%) and 1/60 FN (2%). CGM is potentially more costly to perform than OGTT, with average cost of Medtronic iPro2 = $ 62 AUD and Abbott Freestyle = $ 97 AUD [27, 28]. However, this is unclear as although the Medicare Benefits schedule cost of the OGTT is $19.90 AUD, this is unlikely to reflect the true cost of the test in terms of personnel time involved three blood draws and facilities for patient to wait for 2 h [29]. Regardless, if CGM was shown to be better reflective of women’s glycaemic status than OGTT (i.e., fewer false positives and negatives), it would become cost-saving, given the recent findings of Beilby of costs of almost $6,000 AUD per ante/postnatal care in case of GDM diagnosis (false positive) and over $5,500 per perinatal adverse events in case of not intervention (false negative) [30]. When considering the OGTT/CGM prices and the economic consequences of potential GDM misdiagnosis, the increased cost of CGM appears justified by the potential prospective saving in antenatal and postnatal care. The results of this study encourage the progression in researching the role of CGM for GDM diagnosis to improve women’s experience and potentially HCP’s insight. Due to the limited sample size, deeply affected by the Covid-19 pandemic, this is only to be considered a pilot study. Further research with multicentre studies to explore the correlation between 3 days/7 days of CGM and GDM outcomes in a variety of populations, as well as a detailed OGTT/CGM costs analysis, including the costs of equipment, staff and infrastructures, are needed to strengthen the candidature of CGM as an alternative test to OGTT for GDM diagnosis. We propose that future research could expand this project in terms of acceptability for women as part of a multicentre study on the use of CGM for GDM and in terms of HCP’s feedback with questionnaires to be shared through the national colleges.

### Supplementary Information

Below is the link to the electronic supplementary material.

Supplementary file1 (PDF 735 kb)

Supplementary file2 (PDF 629 kb)

Supplementary file3 (PDF 902 kb)

Supplementary file4 (PDF 908 kb)

## Data Availability

The data that support the findings of this study are not publicly available due to their containing information that could compromise the privacy of research participants but are available from the corresponding author in a de-identified manner upon reasonable request.

## References

[1] Negrato CA, Gomes MB (2013). Historical facts of screening and diagnosing diabetes in pregnancy. Diabetol Metab Syndr.

[2] Bogdanet D (2020). 2020 The oral glucose tolerance test-is it time for a change?-A literature review with an emphasis on pregnancy. J Clin Med.

[3] Kirke AB (2019). Diabetes screening in pregnancy failing women in rural Western Australia: an audit of oral glucose tolerance test completion rates. Aust J Rural Health.

[4] Craig L (2020). Women's experiences of a diagnosis of gestational diabetes mellitus: a systematic review. BMC Pregnancy Childbirth.

[5] Cade TJ, Polyakov A, Brennecke SP (2019). Implications of the introduction of new criteria for the diagnosis of gestational diabetes: a health outcome and cost of care analysis. BMJ Open.

[6] Behboudi-Gandevani S (2019). The impact of diagnostic criteria for gestational diabetes on its prevalence: a systematic review and meta-analysis. Diabetol Metab Syndr.

[7] Hiersch L (2021). DEVELOPING twin-specific 75-g oral glucose tolerance test diagnostic thresholds for gestational diabetes based on the risk of future maternal diabetes: a population-based cohort study. BJOG.

[8] Di Filippo D (2022). Development and evaluation of an online questionnaire to identify women at high and low risk of developing gestational diabetes mellitus. BMC Pregnancy Childbirth.

[9] Trends and Characteristics in Gestational Diabetes: United States, 2016–2020. S. National Center for Health, Editor. Hyattsville, MD. 35877134

[10] Johns EC (2018). Gestational diabetes mellitus: mechanisms, treatment, and complications. Trends Endocrinol Metab.

[11] Simmons D 2019 GDM and Nutrition-Answered and Unanswered Questions-There’s More Work to Do! Nutrients. **11**(8). 10.3390/nu11081940 PMC6722957 31426514

[12] WHO 2020 Mortality and global health estimates.

[13] Lovic D (2020). The growing epidemic of diabetes mellitus. Curr Vasc Pharmacol.

[14] Di Filippo D (2021). The diagnostic indicators of gestational diabetes mellitus from second trimester to birth: a systematic review. Clin Diabetes Endocrinol.

[15] Yu F (2014). Continuous glucose monitoring effects on maternal glycemic control and pregnancy outcomes in patients with gestational diabetes mellitus: a prospective cohort study. J Clin Endocrinol Metab.

[16] Lason I (2021). Continuous glucose monitoring and insulin pump therapy in pregnant women with type 1 diabetes mellitus. Ginekol Pol.

[17] Milln JM (2020). Comparison of oral glucose tolerance test and ambulatory glycaemic profiles in pregnant women in Uganda with gestational diabetes using the FreeStyle Libre flash glucose monitoring system. BMC Pregnancy Childbirth.

[18] Hijazi SAB, Issa H (2010). Continuous glucose monitoring as a diagnostic tool ingestational diabetes. Diab Med..

[19] O'Brien BC (2014). Standards for reporting qualitative research: a synthesis of recommendations. Acad Med.

[20] Australia D gestational diabetes. 2015.

[21] Krzych LJ (2018). The Likert scale is a powerful tool for quality of life assessment among patients after minimally invasive coronary surgery. Kardiochir Torakochirurgia Pol.

[22] Yu Q (2019). Application and utility of continuous glucose monitoring in pregnancy: a systematic review. Front Endocrinol (Lausanne).

[23] Li A, Brackenridge A (2022). The role of continuous glucose monitoring in pregnancy. Obstet Med.

[24] Miller EM (2020). Using continuous glucose monitoring in clinical practice. Clin Diabetes.

[25] Hewapathirana NM, O'Sullivan E, Murphy HR (2013). Role of continuous glucose monitoring in the management of diabetic pregnancy. Curr Diab Rep.

[26] Feig DS (2017). Continuous glucose monitoring in pregnant women with type 1 diabetes (CONCEPTT): a multicentre international randomised controlled trial. Lancet.

[27] Medtronic. iPro2 user guide. 2016. https://www.medtronicdiabetes.com/sites/default/files/library/download-library/user-guides/iPro2-with-Enlite-User-Guide.pdf

[28] Abbott. Introducing the FreeStyle Libre 14 day System. https://www.freestylelibre.com.au/

[29] MBS online. Medicare Benefits Schedule Item 66548. 2023. http://www9.health.gov.au/mbs/fullDisplay.cfm?type=item&q=66548&qt=item

[30] Beilby H (2022). Cost-effectiveness of gestational diabetes screening including prevention of type 2 diabetes: application of the GeDiForCE model in Australia. J Matern Fetal Neonatal Med.

