# Supplementary material for: Oral glucose tolerance test and continuous glucose monitoring for gestational diabetes diagnosis: a survey study of women and health care professionals
Source: Arch Gynecol Obstet. 2023 Feb 5;309(2):483–9. doi: 10.1007/s00404-023-06949-2 (PMC9899442; doi:10.1007/s00404-023-06949-2)
Supplement: Supplementary file 1 — Supplementary file1 (PDF 735 kb) [file 404_2023_6949_MOESM1_ESM.pdf]

Original paper submitted to Acta Diabetologica

# Oral glucose tolerance test and continuous glucose monitoring for gestational diabetes diagnosis: a survey study of women and health care professionals.

*Daria Di Filippo (RM, MBBS) [1], Justine Darling (DE, CNS)[2], Melissa Han Yiin Chang (PHD)[1], Amanda Henry (PHD)[1,3], Alec Welsh (PHD)[1, 4]*

[1] School of Women's and Children's Health
University of New South Wales
Sydney, NSW
Australia

[2] Diabetes Clinic
Royal Hospital for Women
Sydney, NSW
Australia

[3] Department of Women's and Children's Health
St George Hospital
Sydney, NSW
Australia

[6] Department of Maternal-Fetal Medicine
Royal Hospital for Women
Sydney, NSW
Australia

Corresponding author: Professor A W Welsh
Department of Maternal-Fetal Medicine
Royal Hospital for Women
Locked Bag 2000, Barker Street
Randwick, NSW 2031
Australia
Tel: (+61) 293825272
Fax: (+61) 293826444
Email: alec.welsh@unsw.edu.au

Supplementary material 1. CGM and OGTT acceptability questionnaire for women

# CGM acceptability questionnaire -

Please fill this questionnaire to help us understand how pregnant women find CGM.

*Required

1. Email *

2. Name and Surname *

3. Phone number *

4. Did you find the device generally acceptable (tolerable)? *

*Mark only one oval.*

| | 1 | 2 | 3 | 4 | 5 | |
|---|---|---|---|---|---|---|
| I found CGM overall very unacceptable | ⬭ | ⬭ | ⬭ | ⬭ | ⬭ | I found CGM overall very acceptable |

5. Could you please rate on the following scale the acceptability of the insertion of the device: *

*Mark only one oval.*

| | 1 | 2 | 3 | 4 | 5 | |
|---|---|---|---|---|---|---|
| Insertion was very unacceptable | ⬭ | ⬭ | ⬭ | ⬭ | ⬭ | Insertion was very acceptable |

6. Could you please rate on the following scale the acceptability of wearing the device: *

*Mark only one oval.*

| | 1 | 2 | 3 | 4 | 5 | |
|---|---|---|---|---|---|---|
| The device was difficult to wear or caused significant problems | ⬭ | ⬭ | ⬭ | ⬭ | ⬭ | The device was easy to wear and caused no probl[illegible] |

7. Could you please rate on the following scale the acceptability of removal of the device: *

*Mark only one oval.*

| | 1 | 2 | 3 | 4 | 5 | |
|---|---|---|---|---|---|---|
| Removal of the device was very unacceptable | ⬭ | ⬭ | ⬭ | ⬭ | ⬭ | Removal of the device was very acceptable |

8. Would you recommend this form of testing for gestational diabetes to other pregnant women? *

*Mark only one oval.*

| | 1 | 2 | 3 | 4 | 5 | |
|---|---|---|---|---|---|---|
| I would not recommend | ◯ | ◯ | ◯ | ◯ | ◯ | I would recommend this form of testing to other pregnant women |

9. Please provide any further comments in the space below:

_______________________________________________

_______________________________________________

_______________________________________________

_______________________________________________

_______________________________________________

This content is neither created nor endorsed by Google.

Google Forms

# OGTT acceptability questionnaire -

Please fill this questionnaire to help us understand how pregnant women find OGTT.

*Required

1. Email *

2. Name and Surname *

3. Phone number *

4. Did you find the OGTT generally acceptable (tolerable)? *

*Mark only one oval.*

| | 1 | 2 | 3 | 4 | 5 | |
|---|---|---|---|---|---|---|
| I found the OGTT overall very unacceptable | ⬭ | ⬭ | ⬭ | ⬭ | ⬭ | I found the OGTT overall very acceptable |

5. Could you please rate on the following scale the acceptability of having to fast to undergo the test: *

*Mark only one oval.*

| | 1 | 2 | 3 | 4 | 5 | |
|---|---|---|---|---|---|---|
| Fasting was very unacceptable | ⬭ | ⬭ | ⬭ | ⬭ | ⬭ | Fasting was very acceptable |

6. Could you please rate on the following scale the acceptability of the glucose beverage: *

*Mark only one oval.*

| | 1 | 2 | 3 | 4 | 5 | |
|---|---|---|---|---|---|---|
| The glucose beverage was very unacceptable | ⬭ | ⬭ | ⬭ | ⬭ | ⬭ | The glucose beverage was very acceptable |

7. Could you please rate on the following scale the acceptability of blood collection: *

*Mark only one oval.*

| | 1 | 2 | 3 | 4 | 5 | |
|---|---|---|---|---|---|---|
| Blood collection was annoying or caused significant problems | ⬭ | ⬭ | ⬭ | ⬭ | ⬭ | Blood collection was not annoying and caused no p |

8. Could you please rate on the following scale the acceptability of the time frame of the test, including the 2 hours waiting period: *

*Mark only one oval.*

| | 1 | 2 | 3 | 4 | 5 | |
|---|---|---|---|---|---|---|
| The time frame was very unacceptable | ⬭ | ⬭ | ⬭ | ⬭ | ⬭ | The time frame was very acceptable |

9. Would you recommend this form of testing for gestational diabetes to other pregnant women? *

*Mark only one oval.*

| | 1 | 2 | 3 | 4 | 5 | |
|---|---|---|---|---|---|---|
| I would not recommend it | ⬭ | ⬭ | ⬭ | ⬭ | ⬭ | I would recommend this form of testing to other pregnant women |

10. Please provide any further comments in the space below:

______________________________________________

______________________________________________

______________________________________________

______________________________________________

______________________________________________

This content is neither created nor endorsed by Google.

Google Forms
