# Supplementary material for: Oral glucose tolerance test and continuous glucose monitoring for gestational diabetes diagnosis: a survey study of women and health care professionals
Source: Arch Gynecol Obstet. 2023 Feb 5;309(2):483–9. doi: 10.1007/s00404-023-06949-2 (PMC9899442; doi:10.1007/s00404-023-06949-2)
Supplement: Supplementary file 2 — Supplementary file2 (PDF 629 kb) [file 404_2023_6949_MOESM2_ESM.pdf]

Original paper submitted to Acta Diabetologica

Oral glucose tolerance test and continuous glucose monitoring for gestational diabetes diagnosis: a survey study of women and health care professionals.

Daria Di Filippo (RM, MBBS) [1], Justine Darling (DE, CNS)[2], Melissa Han Yiin Chang (PHD)[1], Amanda Henry (PHD)[1,3], Alec Welsh (PHD)[1, 4]

[1] School of Women's and Children's Health
University of New South Wales
Sydney, NSW
Australia

[2] Diabetes Clinic
Royal Hospital for Women
Sydney, NSW
Australia

[3] Department of Women's and Children's Health
St George Hospital
Sydney, NSW
Australia

[6] Department of Maternal-Fetal Medicine
Royal Hospital for Women
Sydney, NSW
Australia

Corresponding author: Professor A W Welsh
Department of Maternal-Fetal Medicine
Royal Hospital for Women
Locked Bag 2000, Barker Street
Randwick, NSW 2031
Australia
Tel: (+61) 293825272
Fax: (+61) 293826444
Email: alec.welsh@unsw.edu.au

Supplementary material 2. Questionnaire for HCP

We would be grateful if you could take the time to complete the following survey, which will take approximately 15 minutes. This survey is a component of a PhD research project, CGM4GDM – Continuous Glucose Monitoring for the diagnosis of Gestational Diabetes Mellitus by Dr Daria Di Filippo, supervised by Professor A. Welsh and A/Prof. A. Henry and funded by the Maternal, Newborn and Women's Health Clinical Academic Group of the Sydney Partnership for Health, Education and Research Enterprise (SPHERE) and UNSW Women Wellbeing Academy (WWBA).

This study seeks to evaluate Continuous Glucose Monitoring (CGM) as an alternative diagnostic test to the currently used OGTT for Gestational Diabetes Mellitus (GDM) and identify the beliefs and attitudes of clinicians (endocrinologists, midwives, obstetricians and diabetic educators) in relation to the current and proposed diagnostic test for GDM.

Our proposal in detail is to:

-Study diagnosis of GDM using 7 days of CGM while women keep track of their diet and exercise routines.

-Evaluate pregnancy outcomes of GDM against CGM parameters and a tailored GDM risk factor versus OGTT.

- Survey both women and clinicians about attitudes regarding GDM diagnostic methods.

Participation to this survey is voluntary and anonymous, and completion of this survey indicates your consent to participate. The findings will be presented to clinicians (medical, midwifery, allied health) in the participating hospitals, and will be presented at conferences or published in academic journals. For more information on this study contact the Principal Investigator, Dr Daria Di Filippo d.difilippo@unsw.edu.au

The conduct of this study at the Royal Hospital for Women and St George Hospital has been approved by the South Eastern Sydney Local Health District (SESLHD) Human Research Ethics Committee. Any person with concerns or complaints about the conduct of this study should contact the Research Support Office which is nominated to receive complaints from research participants. You can contact them on 02 9382 3587, or email SESLHD-RSO@health.nsw.gov.au quoting 2019/ETH04910.

Section 1 - Demographic questions

My discipline/role	◯ Midwife<br>◯ Endocrinologist<br>◯ Diabetic educator<br>◯ Obstetric consultant<br>◯ Obstetric registrar<br>◯ Obstetric resident
My age	◯ 21-30<br>◯ 31-40<br>◯ 41-50<br>◯ 51-60<br>◯ >60<br>◯ Prefer not to say
My years of experience	◯ < 5<br>◯ 5-10<br>◯ 11-15<br>◯ >15<br>◯ Prefer not to say
My primary affiliated public hospital	◯ Royal Hospital for Women<br>◯ St George Hospital

Section 2 - Gestational Diabetes and OGTT

How many patients with GDM do you see a year?

- ◯ < 10
- ◯ 10-25
- ◯ 25-50
- ◯ >50

Do you think GDM has an important impact on public health?

- ◯ Not really
- ◯ Unsure
- ◯ Somewhat important
- ◯ Crucial

Please comment on this

____

Do you have confidence in the OGTT diagnosis?

- ◯ Yes
- ◯ Unsure
- ◯ Not much confidence
- ◯ No confidence at all

Please comment on this

____

Do you see any issue with the current diagnosis of GDM?

- ◯ Not really
- ◯ Unsure
- ◯ Some issues
- ◯ Numerous issues

Please comment on this

____

Do you consider the OGTT generally acceptable for pregnant women?

I consider the OGTT overall very unacceptable — I consider the OGTT overall very acceptable

(Place a mark on the scale above)

Could you please rate on the following scale your perception of the acceptability of the glucose beverage for women?

The glucose beverage is very unacceptable — The glucose beverage is very acceptable

(Place a mark on the scale above)

Could you please rate on the following scale your perception of the acceptability of blood collection for women?

Blood collection is annoying or causes significant problems — Blood collection is not annoying and causes no problems

(Place a mark on the scale above)

Could you please rate on the following scale your perception of the acceptability of the time frame of the OGTT, including the 2 hours waiting period?

The time frame is very unacceptable — The time frame is very acceptable

(Place a mark on the scale above)

Could you please rate on the following scale how reliable you consider the test (correctly giving a true indication of the presence of GDM)?	OGTT is not reliable — OGTT is very reliable<br>*(Place a mark on the scale above)*
Could you please rate on the following scale how replicable you consider the test (likely to give the same result each time if repeated over a short time period)?	OGTT is not replicable — OGTT is very replicable<br>*(Place a mark on the scale above)*
Do you wish to replace this form of testing for gestational diabetes?	I would not wish — I really wish<br>*(Place a mark on the scale above)*

Section 3 - Continuous Glucose Monitor acceptability

Have you ever used a CGM device with your patients?	☐ Never<br>☐ Some of my patients used one but I had no direct experience<br>☐ Yes
Which CGM device have you used?	☐ Medtronic iPro<br>☐ Freestyle Libre<br>☐ Other
Please specify CMG device/s used	__________________________________
Do you consider CGM generally acceptable for patients?	I consider CGM overall very unacceptable — I consider CGM overall very acceptable<br>*(Place a mark on the scale above)*
Could you please rate on the following scale how you find the insertion of the device?	Insertion is very difficult — Insertion is very straightforward<br>*(Place a mark on the scale above)*
Could you please rate on the following scale your perception of the acceptability of the insertion of the device for patients?	Insertion is very unacceptable — Insertion is very acceptable<br>*(Place a mark on the scale above)*
Could you please rate on the following scale your perception of the acceptability of wearing the device for your patients?	The device is difficult to wear or causes significant problems — The device is easy to wear and causes no problems<br>*(Place a mark on the scale above)*
Could you please rate on the following scale how you find the removal of the device?	Removal is very difficult — Removal is very straightforward<br>*(Place a mark on the scale above)*
Could you please rate on the following scale your perception of the acceptability of removal of the device for your patients?	Removal of the device is very unacceptable — Removal of the device is very acceptable<br>*(Place a mark on the scale above)*

Section 4 - Future GDM diagnosis

Would you consider CGM a valid diagnostic tool for GDM?	◯ Not really ◯ Unsure ◯ Probably yes ◯ Absolutely yes
Please comment on this	__________________________________
Do you have experience with any other biomarker of GDM (FBG, SBGM, HbA1c, Fructosamine)?	◯ No ◯ Unsure ◯ Not much experience ◯ Yes
Tests may be combined to optimise diagnostic accuracy, in particular using a technique known as triangulation. We are considering the use of risk factors along with CGM variability to optimise GDM diagnosis. Apart from Risk factors and CGM variability, is there any other parameter you would add to the triangulation?	◯ No ◯ Unsure ◯ Yes
Please comment on this	__________________________________

Please provide any further comments/suggestions for us in the space below.
