# Supplementary material for: Oral glucose tolerance test and continuous glucose monitoring for gestational diabetes diagnosis: a survey study of women and health care professionals
Source: Arch Gynecol Obstet. 2023 Feb 5;309(2):483–9. doi: 10.1007/s00404-023-06949-2 (PMC9899442; doi:10.1007/s00404-023-06949-2)
Supplement: Supplementary file 3 — Supplementary file3 (PDF 902 kb) [file 404_2023_6949_MOESM3_ESM.pdf]

Original paper submitted to Acta Diabetologica

Oral glucose tolerance test and continuous glucose monitoring for gestational diabetes diagnosis: a survey study of women and health care professionals.

Daria Di Filippo (RM, MBBS) [1]*, Justine Darling (DE, CNS)*[2]*, Melissa Han Yiin Chang (PHD)*[1]*, Amanda Henry (PHD)*[1,3]*, Alec Welsh (PHD)*[1, 4]

[1] School of Women's and Children's Health
University of New South Wales
Sydney, NSW
Australia

[2] Diabetes Clinic
Royal Hospital for Women
Sydney, NSW
Australia

[3] Department of Women's and Children's Health
St George Hospital
Sydney, NSW
Australia

[6] Department of Maternal-Fetal Medicine
Royal Hospital for Women
Sydney, NSW
Australia

Corresponding author:

Professor A W Welsh
Department of Maternal-Fetal Medicine
Royal Hospital for Women
Locked Bag 2000, Barker Street
Randwick, NSW 2031
Australia
Tel: (+61) 293825272
Fax: (+61) 293826444
Email: alec.welsh@unsw.edu.au

Supplementary material 4. HCP's free comments on OGTT and CGM

N	Topic	Categories and examples
15	GDM impact	-2x underestimated by women "Women are often very unaware of its significance" "I think the impact GDM has is underestimated as many women have the impression "it will go away"<br>-10 x: long term effect on the health of mothers and babies<br>"GDM has an impact on pregnancy health, postnatal health and future health of the woman, and infant health into the future"<br>-3x impact on healthcare services and obstetric care"<br>"Large impact on service provision, workload, resources if poorly managed"; "overall management, interventions and outcomes and its influence on the obstetric care"; "Even knowing that the mother has GDM alters the obstetric team's thinking regarding trials of instrumental delivery"
14	OGTT limits	-2x pre/analytical/post-analytical issues "The OGTT results depends on tubes used, processing time, inconvenience and current cutoffs are based on observational data rather than proven in rcts of treatment for given cutoffs. It's use early in pregnancy is often misused for gdm diagnosis rather than exclusion of diabetes, and women after bariatric surgery find it difficult and results can be hard to interpret. Having said that, it is currently the best we have"; "Not an individualised test"<br>-7x poor reliability and reproducibility**:** "I have seen so many false positives however more importantly false negatives which is problematic to mum her baby and the team involved in her care", "Overdiagnosis, also different diagnosis criteria depending upon location which is confusing for some health professionals". "GTTs measure values that are thought to be relevant and when composed of a set of arbitrary values are supposed to act as a threshold. Hyperinsulinemia doesn't work like this: there is a continuum of increasing adverse outcomes with increasing blood glucose and/or insulin values. Fixing the time of the routine test at a particular gestation limits sensitivity" "Hard to believe that the one hour value, if the sole abnormality, is a valid diagnostic method.<br>-5x poor acceptability and consideration of the OGTT by patientsAn unpleasant test that women don't want to do"; "Some women don't believe the diagnosis as it's a one-off blood test. Some women think the diagnostic criteria is too strict".
1	New test needed	"Improved ways for diagnosis for pregnant women would be beneficial".
11	Doubts on CGM for GDM	-3x cost/accessibility: ""; "-3x accuracy: "CGM, and especially the Freestyle Libre not being so accurate in extreme BG range especially lower range- we commonly notice underestimation of fasting readings by 1 mmol/L"; """<br>-4x identifying threshold values:<br>-2x lack of evidence:; "lack of evidence for effectiveness in identifying the highest risk patients""; "<br>-2x possibility of bias: "the opportunity for patient to manipulate CGM results by exercising more, eating better, during testing week to avoid GDM diagnosis"; "";
5	Not much knowledge of CGM	"I don't have enough knowledge to judge"; "I would need some training in CGM"
7	CGM advantages	-2x more convenient: "accessible and more convenient than blood samples"; "useful in certain situations including patients who cannot tolerate the GTT or who have had bariatric surgery".<br>-5x more accurate: "dealing with the monitoring data would perhaps provoke a deeper and more nuanced understanding of the disorder"; "more accurate picture and therefore more accurate diagnosis of GDM"; ";"CGM would be a step in the right direction for diagnosing GDM, So many benefits to clients and health professionals"; "I hope this project moves us away from OGTT and starts targeting measures that predict maternal and neonatal/fetal adverse outcome rather than just maternal hyperglycemia".

CGM= continuous glucose monitoring, GDM=gestational diabetes mellitus, HCP= healthcare professionals, OGTT= oral glucose tolerance test
