# Supplementary material for: Oral glucose tolerance test and continuous glucose monitoring for gestational diabetes diagnosis: a survey study of women and health care professionals
Source: Arch Gynecol Obstet. 2023 Feb 5;309(2):483–9. doi: 10.1007/s00404-023-06949-2 (PMC9899442; doi:10.1007/s00404-023-06949-2)
Supplement: Supplementary file 4 — Supplementary file4 (PDF 908 kb) [file 404_2023_6949_MOESM4_ESM.pdf]

Original paper submitted to Acta Diabetologica

Oral glucose tolerance test and continuous glucose monitoring for gestational diabetes diagnosis: a survey study of women and health care professionals.

Daria Di Filippo (RM, MBBS) [1], *Justine Darling (DE, CNS)*[2], *Melissa Han Yiin Chang (PHD)*[1], *Amanda Henry (PHD)*[1,3], *Alec Welsh (PHD)*[1, 4]

[1] School of Women's and Children's Health
University of New South Wales
Sydney, NSW
Australia

[2] Diabetes Clinic
Royal Hospital for Women
Sydney, NSW
Australia

[3] Department of Women's and Children's Health
St George Hospital
Sydney, NSW
Australia

[6] Department of Maternal-Fetal Medicine
Royal Hospital for Women
Sydney, NSW
Australia

Corresponding author: Professor A W Welsh
Department of Maternal-Fetal Medicine
Royal Hospital for Women
Locked Bag 2000, Barker Street
Randwick, NSW 2031
Australia
Tel: (+61) 293825272
Fax: (+61) 293826444
Email: alec.welsh@unsw.edu.au

Supplementary material 3. Women's free comments on OGTT and CGM

N	Topic	Categories and examples
OGTT		
6	Need for an alternative test	"It is very worthwhile to find alternatives to this much complained about test"<br>"It would be preferable not to have to do a test that is such an inconvenience"
1	OGTT over CGM	"The test (OGTT), although time consuming and hard for fasting etc is better in my mind as it is done within 2 hours opposed to waiting a week with the sensor"
2	OGTT not representative of lifestyle	"I would rarely sit for such a long period"<br>"I usually don't eat sugar and control level of carbohydrates in my meals"
2	Impact of OGTT on health	"Feels really unhealthy having the glucose drink", "The idea of putting a large amount of sugar into a body that is fasted is just generally off putting in nature to me when are continually told to avoid sugar/ insulin spikes as they are "bad" for us".
8	Side effect of glucose beverage	"I had sugar high, crash and headache. Was wiped out for most of the day"; "I find the glucose drink very difficult to consume within five minutes, and it makes me feel very nauseated. I experience light-headedness, headache and general unwell feeling afterwards for most of the day" ; "The beverage is horrible and made me sick, especially when you are fasting and experiencing morning sickness"; "The sugar drink caused minor nausea but nothing too concerning".
5	OGTT time frame	"Boring and time consuming", "a big inconvenience" and "quite difficult due to having a toddler". "OGTT is a long process - some people I know have chosen not to complete the OGTT because of the length of time it takes".
4	Importance of GDM screening	"I find it (OGTT) moderately acceptable only due to lack of other options, but acknowledge it is important to identify gestational diabetes early - however it's not an enjoyable process".
CGM		
15	Minimal impact	"I didn't even notice it"; "CGM was quick, easy and painless to insert, caused no trouble during the week I was wearing it, and was easily removed"; "The CGM gave me no discomfort, allowed me to continue living my life normally.
6	CGM over OGTT	"I would absolutely recommend this type of testing for gestational diabetes to the OGTT test, which take a long time to occur and leaves me feeling unwell due to fasting and excessive sugar intake"; "I would recommend this over the OGTT any day"
1	CMG resistance	"Very very impressed at how the device stayed in my arm the entire time - there were even times I forgot it was in and then would be taking off a top, yank it and it would catch the device really hard but it didn't budge - impressed!"
4	Suggestions	-2x shortening CGM period "I would try and get it down to three days of wear. A week is a just a bit of a pain". "A week seems like a long time to wear the device when the current test is only a few hours. I wonder whether an in-between, e.g. 3 days, would give accurate information, too?"<br>-2x self-remove CGM at home: "you should be able to post it back. Coming back for removal is a hassle".
10	Diet tracking problematic	-6xannoying process: "Keeping a food diary for 7 days was sometimes a bit tedious, and I had to keep reminding myself to input what I had eaten and the times"<br>-2xissues with app: "The app that is recommended was extremely frustrating to use"<br>-2x risk bias: "It was good to also try controlling what I ate, to be healthier than usual"<br>"I was monitoring and writing down all good I had and even loss weight which is beneficial"
8	Technical issues with CGM	-2x itchiness: "Device became quite itchy towards the end and left a small itchy red welt after removal"<br>-2x bleeding: "After my insertion I bled a bit through my top and didn't realise therefore stained my top. It bled a second time a few days later minimal"<br>-2xwearing: "My only comment was that the devise rubbed of a tight t shirt may have come loose a day before removal".<br>-2x removal "a little uncomfortable pulling hairs out"

CGM= continuous glucose monitoring, GDM=gestational diabetes mellitus, OGTT= oral glucose tolerance test
